# *Waitea circinata*: a novel biocontrol agent against *Meloidogyne enterolobii* on tomato plants

**DOI:** 10.2478/jofnem-2025-0002

**Published:** 2025-03-14

**Authors:** Gianlucca de Urzêda Alves, CG Felipe, RF Denner, RR Mara, GA Leila

**Affiliations:** Microorganism Genetics Laboratory, Genetics Department, Biological Sciences Institute IV, Universidade Federal de Goiás, Goiânia, Brazil, 74690-900; Nematology Laboratory, Agronomy School, Universidade Federal de Goiás, Goiânia, Brazil, 74690-900

**Keywords:** Biological control, induced resistance, sustainability, root-knot nematode

## Abstract

Root-knot nematodes (RKN), particularly *Meloidogyne enterolobii*, pose a significant threat to agriculture, with current management heavily reliant on agrochemicals due to a scarcity of resistant crop varieties. This study explores orchid mycorrhizae, specifically *Waitea circinata*, as a sustainable biocontrol method to mitigate nematode infestation in tomato plants. The research aimed to compare different application methods and dosages of mycelial suspensions to reduce nematode reproduction and enhance plant health. Two application methods, root immersion for 24 hours and soil drenching, were tested with mycelial suspension concentrations ranging from 5 to 25 g.L^−1^. Results showed that a 15 g.L^−1^ suspension significantly reduced nematode reproduction factor (RF) by 53.4% and nematode density (DENS) by 49.4% while increasing root fresh weight (RFW) by 53.8% in drenching. In subsequent experiments, soil drenching with 15 g.L^−1^ mycelial suspension again reduced RF by 32.41% and DENS by 28.52%, with increases in shoot length (SL) by 26.31%, RFW by 20.42%, and shoot fresh weight (SFW) by 22.20%. Enzymatic analysis revealed that plants treated with *W.circinata* and inoculated with nematodes (Wc+Me) showed a substantial decline in RF (71.13%) and DENS (76.96%). Additionally, there was a marked increase in peroxidase (POX) and catalase (CAT) activity, with Wc plants displaying a 180% increase in POX and a 112.5% increase in CAT at root colonization onset. By day 21, Wc+Me plants exhibited further enzyme activity increases, with POX up by 128% and CAT by 67.6%. This study emphasizes the potential of *W. circinata* in enhancing plant resistance and reducing nematode impact, presenting a promising alternative to chemical control.

Tomato (*Solanum lycopersicum* L.) is a globally important crop, with 186 million tons produced annually on 4.9 million hectares (1). However, productivity fluctuates across regions due to water availability, soil health, and pest pressures (2). In Brazil, which produces approximately 4.1 million tons annually, crop productivity is often limited by biotic stressors such as diseases and pests (3,4). A particularly significant challenge comes from soil-borne nematodes, especially root-knot nematodes (RKNs) of the *Meloidogyne* genus (5).

Several species of *Meloidogyne* threaten tomato crops globally, with *M. javanica* and *M. incognita* being predominant in Brazilian soils (6). However, *M. enterolobii*, an emerging nematode species with a broad host range and the ability to overcome conventional resistance genes, has become a significant concern (7). The management of these nematodes is particularly difficult because the most widely used genetic resistance in tomatoes, based on the Mi gene, fails to suppress *M. enterolobii* populations (8,9).

The available strategies for managing *Meloidogyne* nematodes include chemical control, crop rotation, and resistant cultivars. However, each of these approaches presents significant limitations. Chemical nematicides pose toxicity and environmental risks and offer only temporary suppression; (10) genetic control using resistant crops is ineffective due to *M.enterolobii* virulence; (9) the broad range of hosts and intense use of agricultural systems makes crop rotation economically impractical in many cases (11).

Given the environmental and practical limitations of chemical, genetic, and cultural control strategies, biological control has emerged as a promising alternative for sustainable nematode management. However, the biological control agents currently available in Brazil, including species from the genera *Bacillus*, *Trichoderma*, *Paecilomyces*, and *Pasteuria*, are registered only for controlling *M. incognita* and *M. javanica* (12). No biological control agents are currently registered for *M. enterolobii*, creating a gap in effective nematode management.

Recent research has explored novel biological agents targeting *M. enterolobii*. For instance, fungi species such as *Paraboeremia taiwanensis*, *Samsoniella sp.*, and formulations of *Metarhizium carneum* have shown promise, mainly through egg parasitism (13,14). Despite these developments, further research is needed to expand the diversity of organisms and their practical applications in large-scale tomato production.

This study explores the potential of *Waitea circinata* — a mycorrhizal fungus isolated from native orchids in Brazil — as a biological control agent for *M. enterolobii*. *W. circinata* has demonstrated antagonistic activity against various plant pathogens, including *Magnaporthe oryzae* and *Rhizoctonia solani* (15,16) as well as producing multiple secondary metabolites that were able to control *M. oryzae* development (17). Preliminary studies (unpubl. data) suggest that it not only suppresses *M. javanica* nematode reproduction but may also stimulate plant growth, making it a promising candidate for sustainable nematode management in tomatoes.

We hypothesize that *W. circinata* can serve as a biocontrol agent against *M. enterolobii* by inducing resistance in tomato plants. This study aims to: (i) evaluate the efficacy of *W. circinata* in suppressing the reproduction of *M. enterolobii* populations using different application methods and dosages; (ii) assess the effect of *W. circinata* on plant vegetative traits; (iii) characterize the enzymes associated with induced resistance in tomato plants treated with the most effective biocontrol application method and dosage.

## Materials and Methods

### Study background

The study was conducted in two greenhouses at the Universidade Federal de Goiás, Brazil (Escola de Agronomia and Instituto de Ciências Biológicas IV), located at 16°35′46.4″S 49°16′48.4″W and 16°36′04.4″S 49°15′47.6″W. The climate is classified as Aw (tropical savanna) according to Köppen's classification (18). Greenhouse conditions were maintained with average temperatures of 27 ± 4°C and relative humidity at 70%. The study was conducted between December 2022 and July 2023.

### Experimental design

An experiment was conducted to assess the ability of *W. circinata* to suppress *M. enterolobii* reproduction and its effects on tomato plant growth. It followed a completely randomized factorial design (2 × 6) with two mycorrhizal application methods (immersion and drenching) and six dosages of mycelia (0, 5, 10, 15, 20, and 25 g.L^−1^), using seven replicates per treatment (n = 84 plants per experiment). The experiment was conducted twice, once between December/February 2022–2023 and February/April 2023.

A follow-up experiment assessed the enzymatic activity of the most suppressive treatment identified in the earlier experiments. It used four treatments (Water control, Wc: soil drenching with 15 g.L^−1^ *W. circinata*, Me: inoculation with 2000 eggs + J2 of *M. enterolobii*, and Me+Wc: inoculation with nematodes + 15 g.L^−1^ mycelial suspension). The experiment used six replications for each treatment, with two repetitions of the whole experiment (n = 48 plants).

### Tomato seedling preparation

*Solanum lycopersicum* cv. Santa Cruz Kada seeds, a cultivar susceptible to root-knot nematodes, were sown in 96-cell trays filled with commercial seedling substrate Carolina Soil® (Carolina Soil, Santa Cruz do Sul, Brazil). Fifteen grams of Osmocote® NPK fertilizer (14-14-14) (ICL, São Paulo, Brazil) was applied evenly across the tray. Seedlings were irrigated twice daily and grown under greenhouse conditions for 23 days until they reached 10 cm in height.

### *W. circinata* mycelial suspension preparation

The *W. circinata* isolate (En07) (15) was grown on potato dextrose agar (PDA) plates at 28 ± 2°C for 11 days. Mycelial (0.9 cm diameter) disks were transferred to 40 new PDA plates for further growth. After 12 days, the mycelium was scraped using a sterile scalpel and distributed into Erlenmeyer flasks containing 500 mL of sterilized distilled water to produce the six dosages (0 to 25 g.L^−1^). The suspension was agitated at 150 rpm for 24 hours at 27°C. All procedures were performed in a vertical laminar flow hood sterilized with 70% ethanol and UV irradiation.

### *M. enterolobii* inoculum preparation

The *M. enterolobii* inoculum was obtained from infected sweet potato (*Ipomoea batatas*, cv. Campinense) roots provided by EMBRAPA Clima Tropical, collected in the municipality of Jandaíra, Rio Grande do Norte state (5°17′27″S, 36°06′47″W). This population was kept in sweet potato roots under a greenhouse for inoculum multiplication. The multiplication of *M.enterolobii* was maintained constantly in multiple sweet potato plants on a different section of the greenhouse, with an average of 2–3 months of development to enable multiple nematode life cycles.

The extraction procedure was an adaptation of an established method (19). Infected roots were fragmented into 2 cm pieces and blended with 0.5 % sodium hypochlorite for 30 seconds. The suspension was filtered through 100- and 500-mesh sieves. After washing under running water, the blended root material with water was placed in a 50 mL plastic tube, and 5g of kaolin was added. The filtrate was centrifuged at 1800 rpm for 5 minutes. The pellet was resuspended in 25 mL of a solution of 454 g.L^−1^ of sucrose, then centrifuged at 1800 rpm for 1 minute and filtered in 500-mesh sieves. The collected filtrate was derived from multiple plant samples. A 50 mL aliquot was obtained from the total filtrate volume to count the number of eggs and second-stage juveniles (J2) of *M. enterolobii*. A 1 mL portion of this aliquot was drawn using a pipette and placed into a Peters counting chamber. Observations were conducted under an optical microscope at 40x magnification, and the number of eggs and J2 was counted three times. The final count of eggs and J2 was calculated as the average of these three counts, multiplied by the total volume of the suspension. Subsequently, the suspension concentration was adjusted to contain 1000 eggs and J2 per mL.

### Application methods

The immersion method consisted of taking seedlings with the substrate and immersing the root system completely in 70 mL of the mycelial suspension for 24 hours. After immersion, the seedlings were transplanted into 1L pots filled with a 1:1 sterilized soil-sand mixture pre-autoclaved at 120°C for 40 minutes. The drenching method consisted of taking seedlings, directly transplanting them into 1L pots, and applying 50 mL of the mycelial suspension via soil drenching. All seedlings received 5 g of NPK fertilizer (4-30-16) after transplantation. All plants were irrigated twice daily at 6:00 AM and 6:00 PM using an automated sprinkler system. Seven days post-treatment, the plants were inoculated with 2000 eggs + J2 nematodes per plant.

### Assessment of vegetative and nematode traits

In all experiments, 35 days after nematode inoculation, the following traits were measured: Shoot Length (SL): Measured from the plant base to the last leaf node using a measuring tape; Root Fresh Weight (RFW): Roots were washed, and dried for 1 hour, and weighed using a digital scale (0.001 g precision); Shoot Fresh Weight (SFW): Weighed using the same digital scale; Final Nematode Population (FP): Total nematode count in roots used the method in the extraction for inoculum preparation; Population Density (DENS): Calculated as FP / RFW (eggs + J2 per gram of root) and Reproduction Factor (RF): Calculated as FP / initial inoculum (20).

### Enzymatic activity analysis

Leaves were collected on days 0, 3, 5, 7, 8, 14, and 21 after transplantation and stored in a freezer under −20°C until extraction. For protein extraction, leaf tissue was ground in liquid nitrogen, and 0.1 g of grounded leaves were mixed with an extraction buffer (10 mM Tris-HCl, 150 mM NaCl, 2 mM EDTA, pH 7.0). Samples were centrifuged at 13,000 rpm for 30 minutes at 4°C, and the supernatant was used for enzyme assays.

Peroxidase (POX - EC 1.11.1.7) activity was measured by ABTS (2,2′-azino-bis(3-ethylbenzothiazoline-6- sulfonic acid) oxidation using its colorimetric property at 405 nm, and enzyme activity was defined as the activity that catalyzes the formation of ABTS that increases the absorbance of 1 unit per hour (21). Catalase (CAT - EC 1.11.1.6) activity was measured by the reduction of H_2_O_2_ at 240 nm, and enzyme activity was computed by calculating the amount of H_2_O_2_ decomposed (22). Chitinase (CHI - EC 3.2.1.14) activity was quantified using 3,5-dinitrosalicylic acid (DNS) at 540 nm using colloidal chitin and one unit of enzyme activity was defined as the amount of enzyme needed to release 1 mmol reducing sugar min^−1^ using a modified method (23). All essay readings were conducted on a spectrophotometer.

### Statistical analysis

A Two-way ANOVA and Tukey post-hoc test (p < 0.05) were used to analyze the factorial interaction between application methods and dosages and separate the group means. Each variable residual was analyzed for normality and homoscedasticity using Shapiro-Wilk and Levene's tests. Box-Cox transformations (24) were applied in variables to meet Two-way ANOVA assumptions. A regression analysis was applied for nematological traits (FP, DENS, RF) to find the best descriptor of the dosage effect on nematode reproduction.

Student's t-test was used to compare nematological traits between treatments Me and Me+Wc in the enzymatic experiment to address the consistency of the nematode suppression. Enzyme activities were analyzed using ANOVA with Tukey's test (p < 0.05) to distinguish the group means. Principal component analysis assessed the relationship between enzymatic activity and nematode reproduction factor (RF). All analyses were performed using RStudio (version 4.3.1).

## Results

### Effect of *W. circinata* methods of application and dosages

#### Application method effect

After performing the Two-way ANOVA, no interaction was detected between the application method and dosage for any traits; therefore, the two factors were analyzed independently (Table 1). An analysis of the application method revealed that the drenching method resulted in significantly higher SL, RFW, and SFW than the immersion method (p < 0.05, Table 2). The immersion method exhibited higher population density (DENS), indicating a less developed root system for a similar number of nematodes compared to drenching (p < 0.05, Table 2). The immersion method might affect plant development, reducing vegetative traits compared to the drenching method.

**Table 1:** Analysis of variance of vegetative and nematological variables from tomato plants inoculated with *M.enterolobii* and treated with six concentrations of *W.circinata*, applied as immersion or drench (Experiments 1 and 2).

**Variation Source**	**Dependent Variables^a^**	**Trial 1**		**Trial 2**	
		***F***	***p*-value**	***F***	***p*-value**
Application Method	SL(cm)	1.303^b^	0.257^b^	100.229^b^	<0.001^*^^b^
	RFW(g)	80.338	<0.001^*^	13.596	<0.001^*^
	SFW(g)	0.929	0.338	53.982^b^	<0.001^*^^b^
	FP	1.641^b^	0.208^b^	0.939	0.337
	DENS	2.468^b^	0.120^b^	14.793	<0.001^*^
	RF	1.575^b^	0.213^b^	0.925	0.341

Dosage	SL(cm)	0.636^b^	0.673^b^	1.110^b^	0.368^b^
	RFW(g)	0.151	0.979	0.166	0.973
	SFW(g)	0.906	0.482	1.318^b^	0.272^b^
	FP	2.957^b^	0.017^*^^b^	2.263	0.063
	DENS	2.355^b^	0.048^*^^b^	2.278	0.061
	RF	2.949^b^	0.017^*^^b^	2.256	0.063

Method ^*^ Dosage	SL(cm)	0.524^b^	0.757^b^	0.712^b^	0.617^b^
	RFW(g)	1.317	0.266	1.930	0.106
	SFW(g)	0.960	0.448	1.962^b^	0.101^b^
	FP	0.507^b^	0.769^b^	0.138	0.982
	DENS	0.506^b^	0.770^b^	1.078	0.384
	RF	0.510^b^	0.767^b^	0.081	0.994

a SL = Shoot length; RFW = Fresh root weight; SFW = Fresh weight of aerial part; FP = final population; DENS = Density of nematodes per gram of root; RF = Reproduction Factor.

* The characters have statistically significant differences (p<0.05).

b Analysis carried out with data transformed by the Box-Cox transformations (Box and Cox, 1964).

**Table 2:** Vegetative and nematological traits of the first and second trials, differentiating the application methods of *W. circinata* against *M. enterolobii* in the Santa Cruz Kada tomato cultivar.

		**Immersion**	**Drench**		

**Experiment**	**Trait^a^**	**Mean±SD**	**Mean±SD**	***F***	***p*-value**
1	SL (cm)	110.69 ± 20.01	107.58 ± 15.81	1.303^b^	0.257^b^
	RFW(g)	40.46 ± 10.18	62.26 ± 11.69	80.338	<0.001^*^
	SFW(g)	119.18 ± 29.73	113.88 ± 19.29	0.929	0.338
	FP	1150.00 ± 677.24	1555.95 ± 1187.65	1.641^b^	0.208^b^
	DENS	29.90 ± 18.09	26.06 ± 20.63	2.468^b^	0.120^b^
	RF	0.57 ± 0.33	0.78 ± 0.59	1.575^b^	0.213^b^

2	SL (cm)	90.33 ± 10.11	114.10 ± 8.03	100.229^b^	<0.001^*^^b^
	RFW(g)	29.81 ± 5.77	35.90 ± 7.01	13.596	<0.001^*^
	SFW(g)	91.96 ± 15.30	112.38 ± 7.67	53.982^b^	<0.001^*^^b^
	FP	2443.33 ± 797.27	2248.33 ± 784.38	0.939	0.337
	DENS	92.00 ± 26.37	68.72 ± 23.05	14.793	<0.001^*^
	RF	1.22 ± 0.39	1.12 ± 0.39	0.925	0.341

a SL = Shoot length; RFW = Fresh root weight; SFW = Fresh weight of aerial part; FP = final population; DENS = Density of nematodes per gram of root; RF = Reproduction Factor.

* The characters have statistically significant differences between them (p<0.05).

b Analysis carried out with data transformed by the Box-Cox transformations (Box and Cox, 1964).

#### Dosage effect

The analysis of the dosage factor revealed that the FP, DENS, and FR variables had at least one dosage group mean different from the others (p < 0.05, Table 3). The Tukey test revealed that for FP, DENS, and FR variables, the 15 g.L^−1^ dosage was significantly different from the control treatment (p < 0.05). The regression analysis of the effect of doses of *W. circinata* was best represented by a second-degree polynomial regression for the means of variables FP, RF, and DENS. The regression equation was Y = β_0_ + β_1_X + β_2_X^2^ + ɛ, where Y represents the nematological variable observed (FP, RF, and DENS), and X represents the mycelial suspension dosage. An ANOVA was conducted to evaluate the overall fit of the polynomial regression model, predicting FP, FR, and DENS based on the dosage of mycelial suspension. In both experiments, all models were statistically significant, indicating that the models explained a substantial proportion of the variance in nematological traits (Table 4).

**Table 3:** Vegetative and nematological traits of the first and second trials, differentiating the dosages of *W. circinata* against *M. enterolobii* in the Santa Cruz Kada tomato cultivar.

		**Control (Water)**	**5 g.L^−1^**	**10 g.L^−1^**	**15 g.L^−1^**	**20 g.L^−1^**	**25 g.L^−1^**		

**Experiment**	**Trait^a^**	**Mean±SD**	**Mean±SD**	**Mean±SD**	**Mean±SD**	**Mean±SD**	**Mean±SD**	***F***	***p-value***
1	SL (cm)	101.83 ± 18.79	111.92 ± 13.39	108.64 ± 23.33	107.28 ± 23.35	112.92 ± 13.74	112.21 ± 12.68	0.636^b^	0.673^b^
	RFW(g)	51.31 ± 11.16	52.84 ± 19.36	52.20 ± 17.35	50.33 ± 15.50	51.74 ± 18.11	49.75 ± 12.22	0.151	0.979
	SFW(g)	110.77 ± 21.76	120.73 ± 20.72	117.25 ± 28.44	110.7 ± 27.76	112.88 ± 28.34	126.85 ± 22.37	0.906	0.482
	FP	2053.57 ± 1095.70	1285.71 ± 644.33	1171.42 ± 920.43	967.85 ± 825.46^*^	1203.57 ± 974.37	1435.71 ± 1143.44	2.957^b^	0.017^*^^b^
	DENS	40.59 ± 18.41	27.95 ± 17.37	26.13 ± 24.51	20.23 ± 16.33^*^	24.38 ± 16.26	28.60 ± 19.31	2.355^b^	0.048^*^^b^
	RF	1.02 ± 0.54	0.64 ± 0.32	0.58 ± 0.45	0.48 ± 0.41^*^	0.60 ± 0.48	0.72 ± 0.57	2.949^b^	0.017^*^^b^

2	SL (cm)	107.8 ± 16.51	103.7 ± 16.49	101.1 ± 12.63	100.9 ± 17.16	100.3 ± 14.63	99.5 ± 14.59	1.110^b^	0.368^b^
	RFW(g)	34.27 ± 8.99	33.00 ± 8.04	32.45 ± 5.94	32.10 ± 9.38	32.11 ± 5.20	33.18 ± 5.27	0.166	0.973
	SFW(g)	95.77 ± 25.13	107.27 ± 11.45	106.48 ± 13.21	103.02 ± 17.83	101.01 ± 12.38	99.48 ± 11.20	1.318^b^	0.272^b^
	FP	3015 ± 768.49	2470 ± 825.69	2120 ± 753.58	2035 ± 502.24	2070 ± 727.70	2365 ± 841.64	2.263	0.063
	DENS	95.046 ± 33.35	79.43 ± 21.00	72.47 ± 23.12	67.94 ± 27.72	74.39 ± 27.22	92.90 ± 23.75	2.278	0.061
	RF	1.50 ± 0.38	1.23 ± 0.41	1.06 ± 0.37	1.02 ± 0.25	1.03 ± 0.36	1.18 ± 0.42	2.256	0.063

a SL = Shoot length; RFW = Fresh root weight; SFW = Fresh weight of aerial part; FP = final population; DENS = Density of nematodes per gram of root; RF = Reproduction Factor.

* The characters have statistically significant differences (p<0.05).

b Analysis carried out with data transformed by the Box-Cox transformations (Box and Cox, 1964).

**Table 4:** ANOVA summary for the polynomial regression model of nematological traits based on mycorrhiza mycelial suspension.

			***df***	***SS***	***MS***	***F***	**Significance *F***
Experiment 1	PF^a^	Regression	2	666569.667	333284.834	25.486	^*^0.013
		Residual	3	39231.778	13077.259		
		Total	5	705801.446			
	DENS^a^	Regression	2	223.023	111.512	26.146	^*^0.013
		Residual	3	12.795	4.265		
		Total	5	235.818			
	FR^a^	Regression	2	0.166	0.083	25.698	^*^0.013
		Residual	3	0.010	0.003		
		Total	5	0.176			

Experiment 2	PF^a^	Regression	2	686036.548	343018.274	833.725	^*^0.000
		Residual	3	1234.286	411.429		
		Total	5	687270.833			
	DENS^a^	Regression	2	611.832	305.916	65.484	^*^0.003
		Residual	3	14.015	4.672		
		Total	5	625.847			
	FR^a^	Regression	2	0.171	0.085	888.455	^*^0.000
		Residual	3	0.000	0.000		
		Total	5	0.171			

a FP = final population; DENS = Density of nematodes per gram of root; RF = Reproduction Factor.

* The characters have statistically significant differences between them (p<0.05).

**Figure 1:**
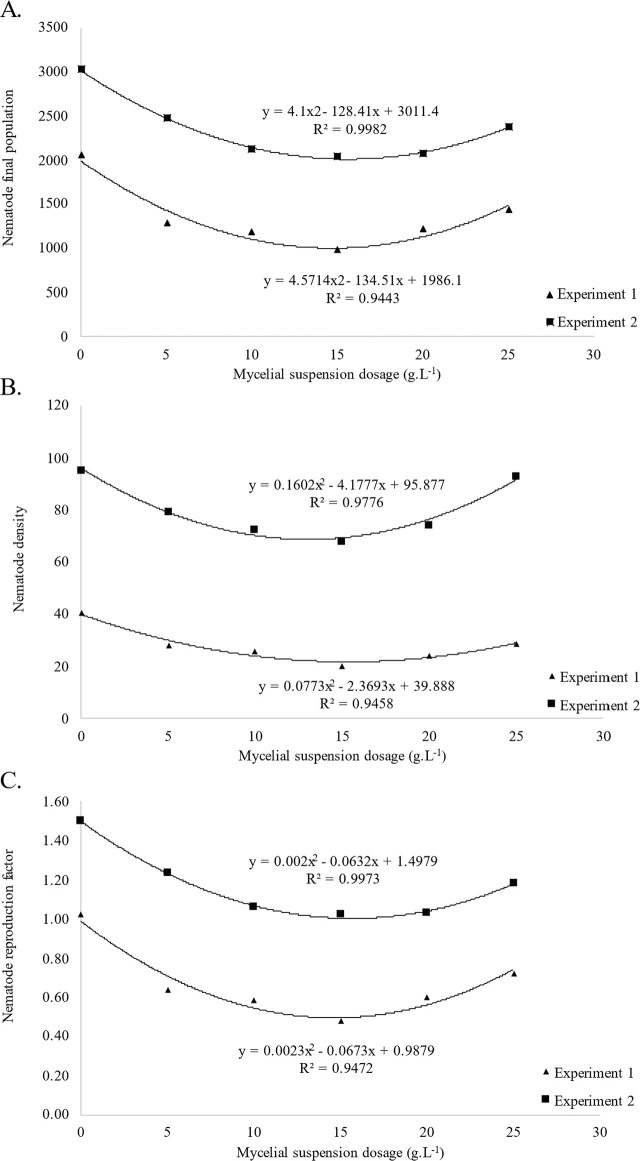
Regression analysis for the effect of *W. circinata* dosages on *M.enterolobii* nematological variables in tomato plants in experiments 1 and 2. A–C) Polynomial regression curves fitted to the means of the final population (FP), density (DENS) and reproduction factor (RF). The data were obtained 35 DAT, and experiments 1 and 2 occurred independently between December 2022 and April 2023.

The regression results indicated that for FP in the first experiment, the 15 g.L^−1^ dosage reduced the control group's average of 2053.57 nematodes to 960.71 nematodes (53.21% reduction). The second experiment lowered from 3015 to 2035 nematodes (32.50% reduction). Based on the equations of the fitted curves, the lowest possible FP obtained by the curves were the dosages of 14.36 and 15.65 g.L^−1^, for experiments 1 and 2, respectively (Fig. 1A).

For DENS, in the first experiment, the 15 g.L^−1^ dosage reduced nematode densities from the control's average of 40.59 nematodes per gram of root to 20.53 (49.42% reduction). In the second experiment, the control average densities were 95.04, lowering to 67.94 nematodes per gram of root (28.51% reduction). The lowest possible DENS values were estimated at 14.71 and 13.03 g.L^−1^ (Fig. 1B).

For RF, when compared to the controls (1.03), the 15 g.L^−1^ dosage obtained the highest suppression, reaching 0.48 in the first experiment (53,39% reduction). In the second experiment, it was 1.51 for the controls, reducing to 1.02 (32.40% reduction). The lowest possible values for RF were estimated to be the dosages of 14.21 and 16 g.L^−1^ on experiments 1 and 2, respectively (Fig. 1C). For the first two experiments, the results converged on the use of dosage of 15g.L^−1^ and the method of soil drenching for enzymatic analysis due to better performance of vegetative traits averages (Tables 2, 3) and *M.enterolobii* population suppression (Figs. 1A–C).

#### Enzymatic activity on tomato plants inoculated with M. enterolobii and treated with W. circinata

The second part of the study focused on analyzing the enzyme activity in plants due to *W. circinata* application and *M. enterolobii* inoculation to determine if systemic resistance was induced. The nematological variables in this experiment evaluated at 35 DAT showed a difference (t-test) between the Me and Wc+Me treatments (treated and non-treated with *W. circinata*) (Table 5). The presence of *W.circinata* in soil drenching in 15 g.L^−1^ dosage resulted in a 71.12% reduction in FP, 76.94% reduction in DENS, and 71.08% reduction in RF when compared with the treatment with *M.enterolobii* only (Table 5).

**Table 5:** Effect of *W. circinata* on nematological variables in tomato plants inoculated with *M. enterolobii* at 35 days after inoculation.

**Treatment**	**FP^a^**	**DENS^a^**	**RF^a^**
Me	2944 ± 668,3	114,43 ± 28,67	1,47 ± 0,33
Wc+Me	850 ± 463,1^*^	26,38 ± 15,41^*^	0,425 ± 0,23^*^

a FP = final population; DENS = Density of nematodes per gram of root; RF = Reproduction Factor.

* The characters have statistically significant differences (p<0.05).

#### POX, CAT, and CHI activity after mycorrhizal application

**Figure 2:**
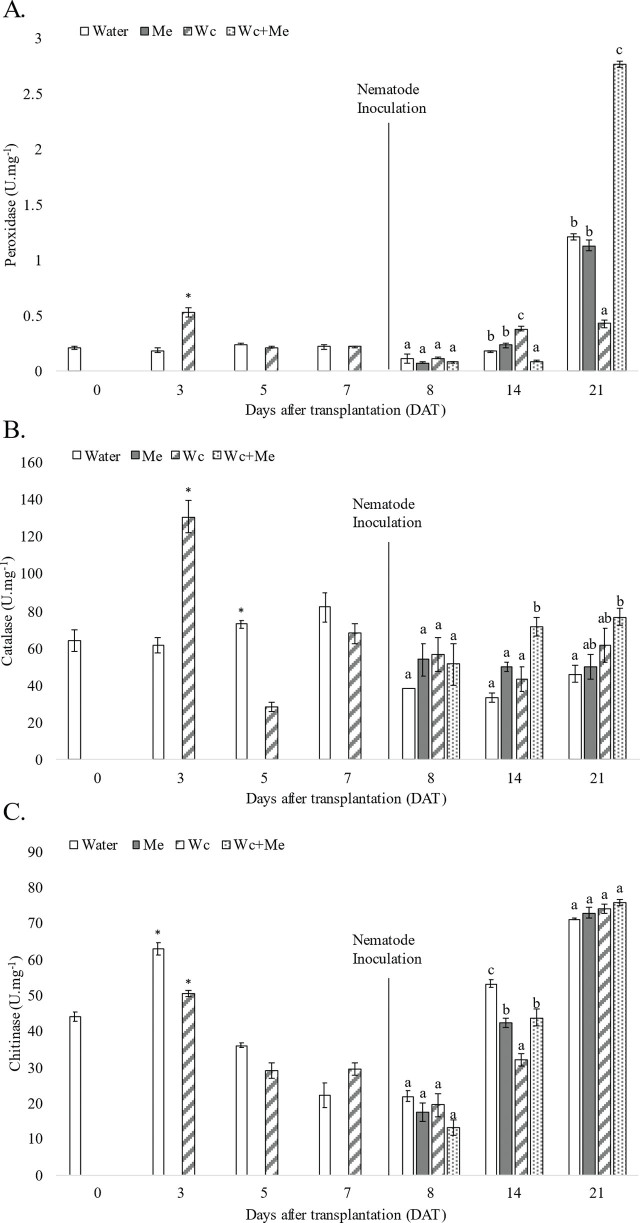
Peroxidase, catalase, and chitinase enzymatic activity analyzed in tomato leaves under different treatments. Negative control (Water), inoculation of 2000 J2 + *M.enterolobii* eggs (Me), irrigation with *W. circinata* mycelial suspension 15 g.L^−1^ (Wc), and treatment with inoculation of 2000 J2 + *M.enterolobii* eggs and watering of *W. circinata* mycelial suspension 15 g.L^−1^ (Wc+Me). Means followed by the same letters in columns were not significantly different according to the Tukey test (*p* < 0.05). Bars indicate the standard error of the mean. A) For POX, T-test revealed a significant difference between enzyme activity between Water (M = 0.190; SD = 0.26) and Wc (M = 0.533; SD = 0.065) on the third day; t(4) = −8.394, *p* = 0.001. B) For CAT, the T-test revealed a significant difference between enzyme activity between Water (M = 61.52; SD = 7.69) and Wc (M = 130.74; SD = 15.38) on the third day; t(4) = −6.971, *p* = 0.002. The T-test revealed a significant difference between enzyme activity between Water (M = 73.06; SD = 3.84) and Wc (M = 28.20; SD = 4.44) on the fifth day; t(4) = 13.229, *p* = 0.000. C) For CHI, the T-test revealed a significant difference between enzyme activity between Water (M = 63.01; SD = 3.11) and Wc (M = 50.39; SD = 1.51) on the third day; t(4) = 6.314, p = 0.003. The T-test revealed a significant difference between enzyme activity (M = 36.15; SD =1.04) and Wc (M = 29.21.74; SD = 3.914) on the fifth day; t(4) = 2.970, *p* = 0.041.

During the first seven days, significant differences in enzyme activity were observed primarily on the third and fifth days after mycorrhizal application. Three days post soil drenching; there was a marked increase in enzyme activity in the treatment with *W. circinata* compared to the control group: POX activity increased 178,94% (Water = 0.19 U.mg^−1^/Wc = 0.53 U.mg^−1^) (Fig. 2A) and CAT activity increased 112,51% (Water = 61.52 U.mg^−1^/Wc = 130.74 U.mg^−1^) (Fig. 2B).

However, CHI activity decreased by 20.02% in the treatment with *W.circinata* (Water = 63.01 U.mg^−1^/Wc = 50.39 U.mg^−1^) (Fig. 2C). On the fifth day, the POX activity showed no differences among treatments. In comparison, CAT and CHI showed a decrease of 96,14% (Water = 73.06 U.mg^−1^/Wc = 28.2 U.mg^−1^) and 19.19% (Water = 36.15 U.mg^−1^/Wc = 29.21 U.mg^−1^) respectively. No more differences between treatments were observed until the nematode inoculation. (Figs. 2A–C).

#### POX, CAT, and CHI activity after M.enterolobii inoculation

All enzyme activities showed differences among treatments on the fourteenth day after mycorrhizal soil drenching and seven days after nematode inoculation. The highest POX activity was recorded for the *W.circinata* (Wc) treatment (0.38 U.mg^−1^), followed by *M.enterolobii* (Me) (0.23 U.mg^−1^), control treatment (0.18 U.mg^−1^) and Wc+Me treatment (0.092 U.mg^−1^). Wc and Wc+Me treatments differed statistically from the control (Fig. 2A). Meanwhile, the highest value for CAT activity was recorded for Wc+Me treatment (71.78 U.mg^−1^). It was the only treatment that differed statistically from the control group (Fig. 2B). The CHI activity was the highest for the control group (53.29 U.mg^−1^), and the lowest for the *W.circinata* treatment (32.22 U.mg^−1^). The Me (42.29 U.mg^−1^) and Wc+Me (43.91 U.mg^−1^) did not differ statistically (Fig. 2C).

On the 21^st^ day after mycorrhizal soil drenching and fourteen days after nematode inoculation POX and CAT activities showed differences among treatments. The Wc+Me treatment (2.76 U.mg^−1^) presented a higher POX activity than the control (1.21 U.mg^−1^). The Me treatment (1.13 U.mg^−1^) showed no differences in POX activity compared to the control group. The Wc treatment (0.42 U.mg^−1^) presented the lowest POX activity of all treatments (Fig. 2A). For CAT activity, the treatment Wc+Me (76.91 U.mg^−1^) showed the highest activity. The Me treatment (49.99 U.mg^−1^) and Wc (61.52 U.mg^−1^) treatments were not different from the control treatment (46.14 U.mg^−1^) (Fig. 2B). No differences were observed for the CHI activity on the 21^st^ day (Fig. 2C).

#### Principal component analysis (PCA)

**Figure 3:**
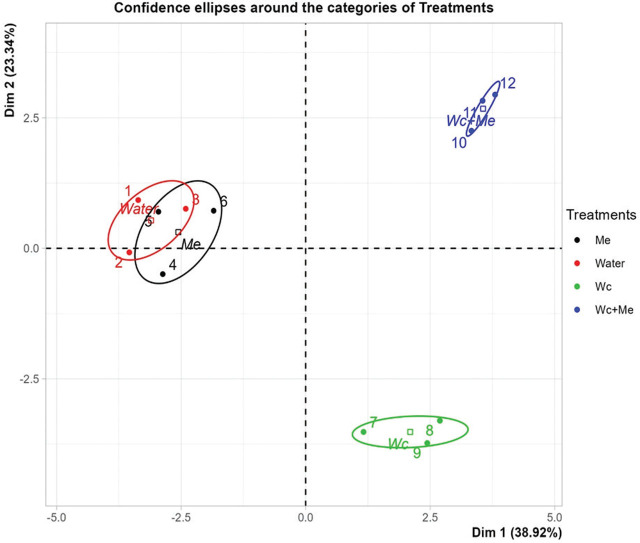
PCA plot of individuals with confidence ellipses around treatment categories and their contribution to Principal Components.

The first two principal components, Dim 1 and Dim 2 explain 38.92% and 23.34% of the total variance, respectively. Confidence ellipses at a 95% confidence level were added to visualize the spread and overlap of the treatment categories. The PCA plot (Fig. 3) displays the samples categorized by treatments, with confidence ellipses around each category. The black (Me) and red (Water) ellipses overlap slightly, indicating some similarity in the samples. Both treatments are clustered near the origin, suggesting limited variance in these treatments along the principal components. Meanwhile, both the green (Wc) and blue (Wc+Me) ellipses are distinctly separated from the other treatments, indicating a unique pattern in those categories (Fig. 3).

**Figure 4:**
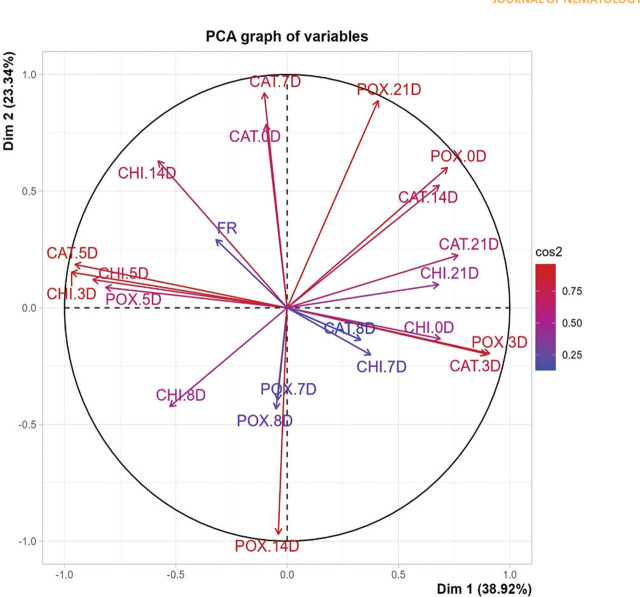
Principal Component Analysis (PCA) plot of enzymatic activities and collection day and their contribution to Principal Components.

The PCA was performed on standardized data to ensure that each variable contributes equally to the analysis. The covariance matrix was computed, and eigenvalues and eigenvectors were extracted to determine the principal components. The vectors’ direction and length indicate each variable's contribution to the principal components. Longer vectors represent variables with higher contributions. The color coding represents the squared cosine (cos2) values, indicating the quality of the representation of the variables on the factor map. Higher cos2 values (red) indicate better representation. CAT.0D, CAT.7D, and POX.21D had longer vectors, indicating a higher contribution to Dim 1 and Dim 2. These variables were key contributors to the variance captured by the first two principal components. Variables related to similar time points or conditions tend to cluster together, suggesting underlying patterns. POX.3D and CAT.3D formed a cluster. Meanwhile, CHI.3D showed an opposite behavior, indicating a strong negative correlation. CAT.5D, CHI.5D, and POX.5D formed a cluster, indicating similar behavior, indicating a strong positive correlation. Both days 7 and 8 showed shorter vectors, except CAT.7D, indicating a lower quality of representation. No clusters were observed on day 14. On day 21, CHI, CAT, and POX showed approximately similar behavior, indicating similar variance characteristics and positive correlation (Fig. 4).

## Discussion

In the present study, we tested two different methods of *W.circinata* mycelia application in tomato plants (drenching and immersion) alongside six different dosages to assess their effects on *M.enterolobii* suppression and plant growth traits in susceptible tomatoes. The findings reveal that soil drenching outperformed immersion in enhancing vegetative traits on tomato plants, increasing RFW in the first experiment and SL, RFW, and SFW in the second experiment. Conversely, immersion showed no statistically significant differences in nematode-related metrics, except for a minor effect on nematode density (DENS) in the second experiment, suggesting that discrepancies in RFW might explain the difference between methods. Since soil drenching also demonstrated greater practicality of application, it appears more suitable for large-scale application in mycorrhizal treatments.

Varying practices in tomato production drove the two-method approach. While soil drenching may suit most field applications, immersion could benefit seedling treatments in pre-planting conditions for fields with known nematode infestations (25). This exploratory investigation into immersion highlights the need for further research to understand its protective effects under different exposure durations. Both methods offer practical value, though cost, labor, and specific crop conditions must be evaluated to optimize applications across different production settings.

The 15 g·L^−1^ mycelial suspension dose was identified as the optimal concentration for suppressing nematode populations, consistently reducing the FP, RF, and DENS across three experiments. An average 50% reduction in FP, RF, and DENS was achieved, suggesting a consistent suppression effect at this dose. Despite conducting trials across two greenhouses at different times of the year, the nematode suppression results remained consistent, supported by regression analysis. This consistency reinforces the efficacy of the 15 g·L^−1^ dosage, although observed variations in suppression intensity may stem from environmental differences inherent in the distinct environmental conditions (26).

Previous studies used *W. circinata* concentrations ranging from 2 to 10 g·L^−1^, typically with *in vitro* co-culture experiments or as foliar sprays to combat fungal pathogens in rice (15,16). These studies found that even autoclaved *W. circinata* mycelia at 5 and 10 g·L^−1^ inhibited colony growth of fungal pathogens, suggesting a potential for heat-resistant bioactive compounds. However, due to the more complex interactions within the soil microbiome, this study expanded the dosage range to determine efficacy under in-soil conditions. Preliminary experiments (unpubl. data) indicated that *W. circinata* could effectively control *M. javanica* at higher dosages, ranging from 15 to 25 g·L^−1^. Consistently, our study identified 15 g·L^−1^ as the most effective dosage for *M. enterolobii* suppression, with higher dosages showing a reduction in efficacy. This decrease may reflect inhibitory effects at excessive dosages, potentially due to altered microbial dynamics, warranting further investigation.

The dose-response relationship between biocontrol agents and pathogens can vary depending on the genetic makeup of the plant, pathogen, and biocontrol agent itself, suggesting that genetic factors may influence the efficacy of mycorrhizal applications. This variability is crucial when developing commercial biocontrol products, as different plant-pathogen systems may require customized dosages for optimal effectiveness (27). The findings from this study provide a foundation for future research aimed at refining dosage guidelines for *M. enterolobii* control, potentially supporting the formulation of targeted biocontrol products. Further research will help establish precise concentration recommendations, thereby enhancing the practical utility of *W. circinata* as a sustainable alternative to chemical nematicides.

The study also explored the enzymatic response associated with *W. circinata* application, focusing on peroxidase (POX), catalase (CAT), and chitinase (CHI) activities. The data suggest that *W. circinata* triggers an oxidative response in tomato plants, marked by increased POX and CAT activity (178.94% and 112.51%, respectively) within the first three days. This heightened oxidative enzyme activity likely reflects an initial stress response due to reactive oxygen species (ROS) production (28–30), which gradually stabilizes by day seven, suggesting a transition into a stable symbiotic state (31,32). The antioxidant enzymes are associated with ROS scavenging, especially during a stressful physiological state, such as an attack by a pathogen (33,34). However, by day five, a correlation between the antioxidant enzymes POX and CAT, which are associated with CHI activity, emerged, indicating potential inter-enzyme interaction in the resistance response.

In the combined treatment (Wc+Me), POX activity spiked by day 21, aligning with 14 days post-nematode inoculation and marking the midpoint of the ideal nematode life cycle (35,36). This response underscores the potential for *W. circinata* to sustain enzymatic defense responses in initial periods, mitigating the exponential growth of the nematode population as infections progress. Additionally, on days 14 and 21, the Wc+Me treatment exhibited higher catalase activity by 115% and 67%, respectively, compared to controls. This heightened CAT activity mirrors findings in other mycorrhizal plant stress responses (37,38) may contribute to a more resilient root environment by neutralizing ROS-induced damage, fostering root health under nematode stress.

Additionally, CHI activity appeared suppressed during initial colonization. This suppression aligns with reports of mycorrhizal fungi, such as *Glomus versiforme*, where CHI activity declines over time, possibly due to the inaccessibility of chitin in fungal cell walls (39,40). The suppression of isoflavonoid phytoalexins defense response in alfalfa roots colonized by *Glomus intraradices* also implies suppression of the host defense system by mycorrhiza (41,42). This selective modulation of CHI activity likely facilitates colonization by minimizing initial plant defenses. However, a positive CHI correlation with RF observed by day 14 suggests that nematode infection may influence or reverse this suppression.

The results parallel findings on other fungal biocontrol agents, such as *Trichoderma* spp. and *Purpureocillium lilacinum*, known for their multifaceted mechanisms against nematodes, including egg parasitism, juvenile antagonism, and plant-induced resistance (43,44). *Trichoderma harzianum* manages *M. javanica* in tomatoes through a similar suite of lytic enzymes, fungal metabolites, and induced resistance. The present study suggests that *W. circinata* could operate through comparable mechanisms, especially induced resistance (45). Additionally, parallels with *Purpureocillium lilacinum*, which parasitizes nematode eggs and juveniles, suggest that *W. circinata* may exert direct antagonism (46). However, despite observing egg parasitism during nematode extraction and counting, this study did not quantitatively measure this effect, marking a need for future research on the extent and conditions favoring egg parasitism by *W. circinata*.

The study highlights W. circinata's potential as a biocontrol agent against root-knot nematodes, paving the way for further research on their application and resistance induction mechanisms. By elucidating these dynamics, we aim to enhance sustainable agricultural practices that reduce reliance on chemical treatments and improve plant resilience through symbiotic fungal associations.

To fully understand *W. circinata*'s biocontrol potential, future research should examine enzyme activity profiles across extended interaction periods to capture long-term resistance induction trends; investigate how environmental variables such as temperature, humidity, and soil type may influence application efficacy and enzyme response in field conditions.

## Conclusion

Our study demonstrated that soil drenching with *W. circinata* mycelial suspension at a 15 g·L^−1^ dosage effectively suppressed *M. enterolobii* populations in tomato plants, achieving a 50% reduction in nematode multiplication and increasing key vegetative growth metrics—stem length (SL), root fresh weight (RFW), and shoot fresh weight (SFW)—by an average of 20% compared to the immersion method. Enzymatic analyses indicated early root colonization by *W. circinata*. A priming effect was sustained, as evidenced by positive correlations among enzymatic activities from days 14 to 21, suggesting an enhanced stress response. Specifically, mycorrhized plants produced higher levels of antioxidant enzymes in response to nematode infestation, indicating an adaptive mechanism to manage oxidative stress induced by *M. enterolobii*.

The suppression rates achieved in this study are comparable to other biocontrol studies involving arbuscular mycorrhizal fungi (AMFs), highlighting *W. circinata* as a viable alternative for *M. enterolobii* biocontrol in tomato cultivation. These findings contribute to the growing body of research on sustainable biological controls, underscoring the potential of *W. circinata* as an eco-friendly, effective agent against root-knot nematodes. As agricultural systems increasingly shift towards sustainable practices, *W. circinata* offers a promising tool for integrated pest management strategies that reduce reliance on chemical nematicides and enhance plant resilience through symbiotic associations. Further research will be essential to refine application techniques and dosage recommendations, supporting broader adoption in commercial tomato production.
